# Association of Arterial Metabolic Content with Cerebral Blood Flow Regulation and Cerebral Energy Metabolism–A Multimodality Analysis in Aneurysmal Subarachnoid Hemorrhage

**DOI:** 10.1177/08850666221080054

**Published:** 2022-02-16

**Authors:** Teodor Svedung Wettervik, Anders Hånell, Timothy Howells, Elisabeth Ronne-Engström, Per Enblad, Anders Lewén

**Affiliations:** 1 8097 Uppsala University, Uppsala, Sweden

**Keywords:** aneurysmal subarachnoid hemorrhage, arterial blood gas, cerebral microdialysis, cerebral pressure reactivity, neurointensive care

## Abstract

**Background:**

In this study, the association of the arterial content of oxygen, carbon dioxide, glucose, and lactate with cerebral pressure reactivity, energy metabolism and clinical outcome after aneurysmal subarachnoid hemorrhage (aSAH) was investigated.

**Methods:**

In this retrospective study, 60 patients with aSAH, treated at the neurointensive care (NIC), Uppsala University Hospital, Sweden, between 2016 and 2021 with arterial blood gas (ABG), intracranial pressure, and cerebral microdialysis (MD) monitoring were included. The first 10 days were divided into an early phase (day 1 to 3) and a vasospasm phase (day 4 to 10).

**Results:**

Higher arterial lactate was independently associated with higher/worse pressure reactivity index (PRx) in the early phase (β = 0.32, *P* = .02), whereas higher pO_2_ had the opposite association in the vasospasm phase (β = −0.30, *P* = .04). Arterial glucose and pCO_2_ were not associated with PRx. Higher arterial lactate (β = 0.29, *P* = .05) was independently associated with higher MD-glucose in the vasospasm phase, whereas higher pO_2_ had the opposite association in the vasospasm phase (β = −0.33, *P* = .03). Arterial glucose and pCO_2_ were not associated with MD-glucose. Higher pCO_2_ in the early phase, lower arterial glucose in both phases, and lower arterial lactate in the vasospasm phase were associated (*P* < .05) with better clinical outcome.

**Conclusions:**

Arterial variables associated with more vasoconstriction (higher pO_2_ and lower arterial lactate) were associated with better cerebral pressure reactivity, but worse energy metabolism. In severe aSAH, when cerebral large-vessel vasospasm with exhausted distal vasodilation is common, more vasoconstriction could increase distal vasodilatory reserve and pressure reactivity, but also reduce cerebral blood flow and metabolic supply. The MD may be useful to monitor the net effects on cerebral metabolism in PRx-targeted NIC.

## Introduction

After aneurysmal subarachnoid hemorrhage (aSAH), the brain is very susceptible to secondary insults that may lead to secondary brain injury. Neurointensive care (NIC) aims at optimizing the cerebral environment and improve outcome by preventing, detecting, and treating these secondary insults.^1–4^ Optimal cerebral delivery of oxygen and energy metabolic substrates is key for neuronal survival after aSAH. This delivery depends on cerebral blood flow (CBF) and the arterial content of oxygen and energy metabolites. CBF is often disturbed and ischemia is common in severe aSAH, particularly day 4 to 10, due to a combination of large-vessel vasospasm, loss of cerebral pressure reactivity, and too low cerebral perfusion pressure (CPP).^5,67,8^ Current management aims at maintaining a sufficiently high CPP to prevent and occasionally treat delayed ischemic neurological deficits (DIND).^1,2^

Likewise, optimal arterial content of oxygen and energy substrates are important to prevent DIND. Higher content of these variables could compensate for ischemic CBF, but more is not always better. There is some support for arterial hyperoxia treatment in traumatic brain injury (TBI),^9,10^ but higher pO_2_ may induce cerebral vasoconstriction^11,12^ and reactive oxygen species^13^ and is also associated with cerebral vasospasm,^14^ DIND,^15,16^ and unfavorable outcome^15,16^ in aSAH. Furthermore, pCO_2_ is a metabolic end-product and high levels lead to cerebral vasodilation and increase CBF. Hyperventilation is used to treat high intracranial pressure (ICP) and possibly improve cerebral pressure reactivity in TBI,^17,18^ but normo-/hypercapnia may counteract ischemic CBF,^19^ increase brain tissue oxygenation,^19^ reduce DIND,^20^ and improve clinical outcome in aSAH.^20,21^ Both glucose and lactate are substrates in cerebral energy metabolism.^22^ Hypoglycemia predisposes for neuroglucopenia, but hyperglycemia is also detrimental and may disturb cerebral pressure reactivity^23,24^ and oxidative energy metabolism^24^ in TBI, and correlates with worse outcome in both TBI and aSAH.^24,25^ Lactate is an alternative cerebral energy source to glucose^22^ and intravenous supplement in acute brain injury could spare cerebral glucose.^26^ Lactate also works as a vasodilator and could increase CBF,^27^ but possibly at the expense of more disturbed cerebral pressure reactivity.^28^

Hence, the regulation of and the absolute CBF are together with the arterial metabolic content important variables to meet brain energy metabolic demand. These variables exhibit a complex interplay and although they are key targets in NIC protocols,^29^ there is limited knowledge on the net effect on the brain following NIC interventions that manipulate one or many of these variables. To gain further understanding, more studies of CBF pressure autoregulation and cerebral energy metabolism based on multimodality data of cerebral physiology are needed.

In this study, the primary aim was to investigate the association among the arterial metabolic content variables pO_2_, pCO_2_, glucose, and lactate with the cerebral pressure reactivity and energy metabolism, respectively, in severe aSAH. The secondary aim was to study these variables in relation to DIND and clinical outcome.

## Materials and Methods

### Patients

In this retrospective study, patients with aSAH admitted to the Department of Neurosurgery at the University Hospital in Uppsala, Sweden, November 2016 to May 2021, were eligible for this study. Out of 561 SAH patients, we included those 60 aSAH patients who had ICP, arterial blood gas (ABG) and microdialysis (MD) monitoring data.

### Treatment Protocol

Patients were treated in accordance with our standardized treatment protocol to avoid secondary insults, which has been described in detail in previous studies.^4,30,31^ Treatment goals were ICP ≤ 20 mm Hg, CPP ≥ 60 mm Hg, systolic blood pressure > 100 mm Hg, pO_2_ > 12 kPa, normoventilation, arterial glucose 5–10 mmol/L (mM), electrolytes within normal ranges, slight hypervolemia after aneurysm occlusion, and body temperature < 38 °C. Patients not responding to command was mechanically ventilated and received a ventricular catheter system for ICP monitor and drainage of cerebrospinal fluid (CSF) when needed.

DIND was clinically defined, as new focal neurological deficits and/or decreased level of consciousness when other causes, eg hydrocephalus and hematomas, were excluded. The treatment of DIND was HHH (hypertension, hypervolemia, and hemodilution)-therapy was initiated with 200 ml albumin (200 mg/ml) intravenously administered for 5 days.

### Data Acquisition and Analyses

ICP was monitored with an external ventricular drainage (EVD) system (HanniSet, Xtrans, Smith Medical GmbH, Glasbrunn, Germany or VentrEX, Neuromedex, Hamburg, Germany). Arterial blood pressure (ABP) was monitored in the radial artery at heart level. Physiological data were collected at 100 Hz using the Odin software.^32^ Pressure reactivity index (PRx) was continuously calculated as the 5 min correlation of 10 s averages of ICP and MAP.^33,34^ ABG data were analyzed in samples taken through the radial arterial line every fourh hour, more often if needed. ABG samples were analyzed on an ABL800 FLEX instrument (Radiometer, Copenhagen).

Cerebral energy metabolism was monitored with the 70 High Cut-Off MD catheter with a membrane length of 10 mm and a membrane cut-off of 20 kDa (M Dialysis AB, Stockholm, Sweden). The MD catheter was placed adjacent to the EVD in normal-appearing brain tissue in the right frontal lobe. The MD was perfused by means of a microinjection pump (106 MD Pump, M Dialysis AB) at a rate of 0.3 µL/min with custom made sterile artificial CSF containing – NaCl 147 mmol/L (mM), KCl 2.7 mM, CaCl_2_ 1.2 mM, and MgCl_2_ 0.85 mM. Cerebral interstitial glucose, pyruvate, lactate, and urea were analyzed hourly, using a CMA 600 analyzer or the ISCUSflex Microdialysis Analyzer (M Dialysis AB). The MD urea was monitored to validate catheter performance.^35^

### Outcome

Clinical outcome was evaluated according to the Extended Glasgow Outcome Scale (GOS-E)^36,37^ 12 months after ictus, by trained personnel using structured telephone interviews. GOS-E has eight categories of outcome that ranges from death (1) to upper good recovery (8).

### Statistical Analysis

The analysis primarily aimed to determine the association of the four ABG variables (pO_2_, pCO_2_, arterial glucose, and arterial lactate) with cerebral pressure reactivity (PRx) and energy metabolism (MD-glucose, MD-pyruvate, MD-lactate, and MD-LPR) (i) and secondarily to determine the association of these ABG variables with DIND and clinical outcome (GOS-E) (ii).

Nominal, ordinal, and continuous variables were described as numbers or proportions, medians (interquartile range [IQR]), and means (±standard deviation [SD]), respectively. The first 10 day period post-ictus was divided into two phases – (a) Early phase (day 1 to 3) and (b) Vasospasm phase (day 4 to 10). Mean values for the physiological variables mentioned above were calculated for each phase. These physiological analyses were done in the Odin software^32^ and the data were then transferred to SPSS version 27 (IBM Corp, Armonk, NY, USA) for further statistical analyses.

Paired t-tests were done to determine if there were any changes in the physiological variables from the early phase to the vasospasm phase. The associations of the four ABG variables in relation to cerebral pressure reactivity (PRx) and cerebral energy metabolism (MD-glucose, MD-pyruvate, MD-lactate, and MD-lactate-pyruvate ratio [LPR]) were evaluated using univariate analyses (Spearman) for both phases. Multiple linear regression analyses for the early phase and the vasospasm phase were performed with PRx as the dependent variable and age, GCS M, and CPP as baseline variables in addition to the significant variables from the univariate analyses (pO_2_ and arterial lactate). Similar regressions were performed with MD-glucose as the dependent variable and the same independent variables as for the regressions with PRx. Those with missing values were excluded from the analyses.

The relation of the four ABG variables with DIND and clinical outcome (GOS-E) was analyzed with the Mann-Whitney U-test and the Spearman correlation test, respectively.

A *P*-value < .05 was considered statistically significant.

### Ethics

All procedures performed in the studies involving humans were in accordance with the ethical standards of the institutional and national research committee and with the 1964 Helsinki declaration and its later amendments. The study was approved by the regional ethical board (2010/138 and 2010/138/1) and the Swedish Ethical Review Authority (2020-05462). Written informed consent was obtained by the closest relatives or from the patients after recovery. In those cases when the relatives or the patients had not responded at follow-up, consent was waived if the relatives or the patient had not objected to take part in the study (2020-05462).

## Results

### Demography, Admission Variables, Treatments, and Clinical Outcome

Sixty patients were included, of whom 44/16 (73/27%) were female/male and mean age was 57 ± 12 years (Table 1). Glasgow Coma Scale Motor (GCS M) score was in median 5 (interquartile range [IQR] 5-6), World Federation of Neurosurgical Societies (WFNS) grade was IV-V in 42 (70%) patients, no patient had abnormal pupillary light reactions at admission, and Fisher grade was in median 4 (IQR 3-4). The aneurysm was in the anterior/posterior circulation in 48/12 (80/20%) cases and 53 (88%) were treated with endovascular embolization, 6 (10%) with clipping and 1 (2%) patient received no aneurysm treatment. Furthermore, 19 (32%) patients developed DIND, 3 (5%) received thiopental infusion, and 3 (5%) decompressive craniectomy. Seven (16%) patients died and GOS-E was in median 4 (IQR 3-6) after 1 year.

**Table 1.** Demography, Admission Variables, Treatments, and Clinical Outcome After Aneurysmal Subarachnoid Hemorrhage.

Patients, n (%)	60 (100%)
Age, mean ± SD	57 ± 12
Sex (female), n (%)	44 (73%)
GCS M, median (IQR)	5 (5-6)
WFNS grade IV-V, n (%)	42 (70%)
Pupillary abnormality, n (%)	0 (0%)
Fisher grade, median (IQR)	4 (3-4)
Aneurysm (anterior/posterior), n (%)	48/12 (80/20%)
Aneurysm treatment (embolization/clipping/no), n (%)	53/6/1 (88/10/2%)
DIND (yes), n (%)	19 (32%)
Thiopental, n (%)	3 (5%)
DC, n (%)	3 (5%)
GOS-E, median (IQR)*	4 (3-6)
Mortality, n (%)*	7 (16%)

DC = Decompressive craniectomy, DIND = Delayed ischemic neurological deficit, GCS M = Glasgow Coma Scale Motor score, GOS-E = Glasgow Outcome Scale Extended, IQR = Interquartile range, SD = Standard deviation, WFNS = World Federation of Neurosurgical Societies.

*17 patients with missing data.

### Physiological Variables in the Early Phase and the Vasospasm Phase

The physiological variables are described in Table 2. From the early phase to the vasospasm phase, mean pO_2_ decreased slightly from 15 ± 2 to 13 ± 1 kPa (*P*-value = .001), pCO_2_ remained stable from 5.1 ± 0.4 to 5.2 ± 0.5 kPa (*P*-value = .27), arterial glucose was also stable from 8.7 ± 1.3 to 8.5 ± 1.0 mM (*P*-value = .08), and arterial lactate decreased from 1.6 ± 0.5 to 1.2 ± 0.3 mM (*P*-value = .001). Furthermore, ICP remained at 12 ± 3 mm Hg in both phases (*P*-value = .48), CPP increased from 76 ± 6 to 82 ± 9 mm Hg (*P*-value = .001), and PRx was unchanged from 0.13 ± 0.13 to 0.15 ± 0.13 (*P*-value = .25). For the MD-variables, MD-glucose changed from 2.4 ± 1.4 to 2.6 ± 1.5 mM (*P*-value = .40), MD-pyruvate increased from 122 ± 37 to 158 ± 48 µM (*P*-value = .001), MD-lactate increased from 4.3 ± 2.3 to 4.9 ± 2.0 (*P*-value = .002) and MD-LPR decreased from 38 ± 22 to 31 ± 10 (*P*-value = .02).

**Table 2.** Systemic and Intracranial Physiological Variables in the Early Phase and the Vasospasm Phase After Aneurysmal Subarachnoid Hemorrhage.

Variables	Early phase	Vasospasm phase	p-value
pO_2_ (kPa), mean ± SD	15 ± 2	13 ± 1	0.001
pCO_2_ (kPa), mean ± SD	5.1 ± 0.4	5.2 ± 0.5	0.27
Arterial glucose (mM), mean ± SD	8.7 ± 1.3	8.5 ± 1.0	0.08
Arterial lactate (mM), mean ± SD	1.6 ± 0.5	1.2 ± 0.3	0.001
ICP (mm Hg), mean ± SD	12 ± 3	12 ± 3	0.48
CPP (mm Hg), mean ± SD	76 ± 6	82 ± 9	0.001
PRx (coefficient), mean ± SD	0.13 ± 0.13	0.15 ± 0.13	0.25
MD-glucose (mM), mean ± SD	2.4 ± 1.4	2.6 ± 1.5	0.40
MD-pyruvate (µM), mean ± SD	122 ± 37	158 ± 48	0.001
MD-lactate (mM), mean ± SD	4.3 ± 2.3	4.9 ± 2.0	0.002
MD-LPR (coefficient), mean ± SD	38 ± 22	31 ± 10	0.02

CPP = Cerebral perfusion pressure, ICP = Intracranial pressure, LPR = Lactate-pyruvate ratio, MD = Microdialysis, PRx = Pressure reactivity index, SD = Standard deviation.

### Arterial Blood gas Variables in Relation to Cerebral Pressure Autoregulation

**Figure 1.**
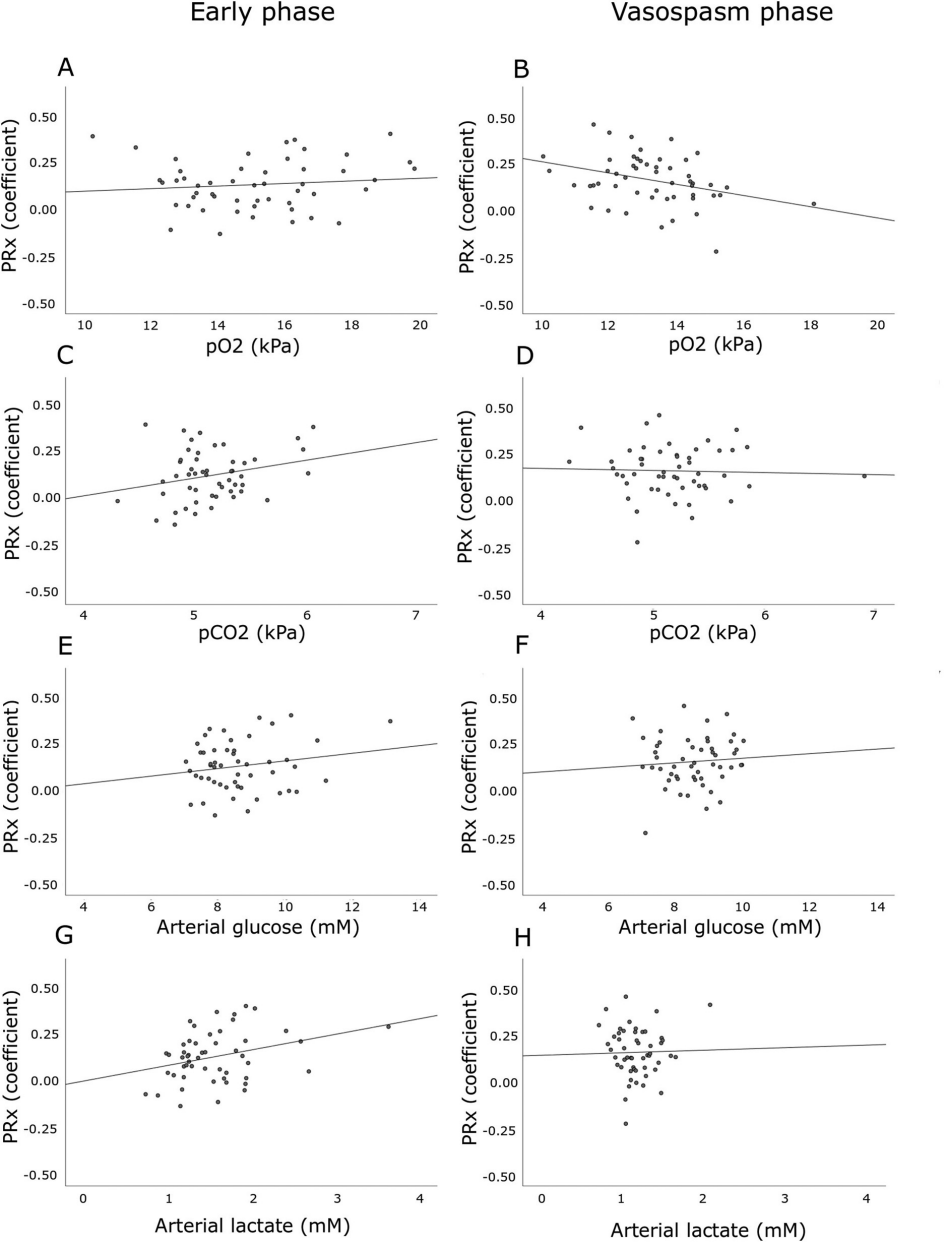
(A–H). Arterial blood gas variables in relation to cerebral pressure autoregulation in the early phase and the vasospasm phase after aneurysmal subarachnoid hemorrhage. The figures demonstrates the associations of PRx with pO_2_ (1A-B), pCO2 (2C-D), arterial glucose (2E-F), and arterial lactate (2G-H) in the early phase and the vasospasm phase. Higher pO_2_ in the vasospasm phase (r = −0.32, *P* < .05) and lower arterial lactate in the early phase (r = 0.31, *P* < .05) were significantly associated with lower PRx.

Higher arterial lactate in the early phase, but not in the vasospasm phase, correlated with higher PRx (Table 3, Figure 1). There was no association between pO_2_ and PRx in the early phase, but higher pO_2_ correlated with lower PRx in the vasospasm phase. Otherwise, pCO_2_ and arterial glucose were not associated with PRx in any of the two phases. In multiple linear regression analyses with PRx as the dependent variable (Table 4), higher arterial lactate was independently associated with higher PRx in the early phase and higher pO_2_ was independently associated with lower PRx in the vasospasm phase, similar to the univariate analyses.

**Table 3.** Arterial Blood gas Variables in Relation to Cerebral Pressure Autoregulation, Cerebral Energy Metabolism, and Clinical Outcome in the Early Phase and the Vasospasm Phase After Aneurysmal Subarachnoid Hemorrhage – a Spearman Rank Correlation Analysis.

	**pO_2_**		**pCO_2_**		**Arterial glucose**		**Arterial lactate**	
	Early phase (r)	Vasospasm phase (r)	Early phase (r)	Vasospasm phase (r)	Early phase (r)	Vasospasm phase (r)	Early phase (r)	Vasospasm phase (r)
PRx	0.11	***−0.32^a^***	0.19	−0.02	0.05	0.10	***0.31^a^***	−0.08
MD-glucose	***−0.29^a^***	***−0.36^a^***	0.09	−0.03	0.26	0.21	−0.05	***0.28^a^***
MD-pyruvate	−0.01	0.02	−0.11	−0.09	−0.07	0.19	−0.10	0.06
MD-lactate	0.03	0.07	−0.03	−0.01	−0.19	0.20	−0.19	−0.06
MD-LPR	−0.01	0.14	0.03	0.27	−0.16	0.07	−0.17	−0.01
GOS-E	0.00	−0.13	***0.38^a^***	0.01	***−0.40^b^***	***−0.40^b^***	−0.22	***−0.41^b^***

^a^ *P*-value < .05 ^b^*P*-value < .01.

ABG = Arterial blood gas, GOS-E = Glasgow Outcome Scale Extended, LPR = Lactate-pyruvate ratio, MD = Microdialysis, PRx = Pressure reactivity index.

Correlation analyses between ABG-variables and PRx: Early phase, n = 51. Late phase, n = 50.

Correlation analyses between ABG- (pO2, pCO2, arterial glucose, and arterial lactate) with MD-variables (MD-glucose, MD-pyruvate, MD-lactate, and MD-LPR): Early phase, n = 51. Vasospasm phase, n = 48.

Correlation analyses between ABG-variables and GOS-E: Early phase, n = 40. Late phase, n = 42.

**Table 4.** Prediction of Pressure Reactivity index and Cerebral Glucose in the Early Phase and the Vasospasm After Aneurysmal Subarachnoid Hemorrhage – Multiple Linear Regression Analyses.

PRx				
Variables	Early phase *(i)*		Vasospasm phase *(ii)*	
	β (95% CI)	p-value	β (95% CI)	p-value
Age	−0.03 (-1.20-0.90)	0.81	0.03 (-0.10-0.14)	0.81
GCS M	0.32 (0.05-0.60)	***0.02***	0.19 (-0.09-0.45)	0.17
CPP	-0.09 (-0.35-0.17)	0.55	-0.26 (-0.52-0.00)	0.07
pO_2_	0.09 (-0.17-0.36)	0.50	-0.30 (-0.57-0.02)	***0.04***
Arterial lactate	0.32 (0.05-0.59)	***0.02***	-0.02 (-0.30-0.26)	0.90
**MD-glucose**				
Variables	Early phase *(iii)*		Vasospasm phase *(iv)*	
	β (95% CI)	p-value	β (95% CI)	p-value
Age	-0.02 (-0.26-0.23)	0.89	-0.35 (-0.66 - -0.05)	***0.03***
GCS M	-0.06 (-0.36-0.23)	0.68	0.02 (-0.27-0.31)	0.89
CPP	-0.03 (-0.36-0.29)	0.83	-0.23 (-0.52-0.06)	0.12
pO_2_	-0.31 (-0.61-0.00)	***0.05***	-0.33 (-0.62- - -0.04)	***0.03***
Arterial lactate	-0.03 (-0.32-0.27)	0.86	0.29 (0.00-0.57)	***0.05***

Regression of PRx as the dependent variable in the early phase *(i)*, R^2^ = 0.21, ANOVA, *P*-value = .05, n = 50, and in the vasospasm phase *(ii)*, R^2^ = 0.21, ANOVA, *P*-value = .05, n = 51. Regression of MD-glucose as the dependent variable in the early phase *(iii)*, R^2^ = 0.09, ANOVA, *P*-value = .50, n = 48, and in the vasospasm phase *(iv)*, R^2^ = 0.24, ANOVA, *P*-value = .04, n = 47.

CI = Confidence interval, CPP = Cerebral perfusion pressure, GCS M = Glasgow Coma Scale Motor Score, MD = Microdialysis, PRx = Pressure reactivity index.

### Arterial Blood gas Variables in Relation to Cerebral Energy Metabolism

Higher pO_2_ was associated with lower MD-glucose in both the early phase and the vasospasm phase in univariate analyses (Table 3). Higher arterial lactate was associated with higher MD-glucose in the vasospasm phase. These two ABG variables were otherwise not associated with the other MD-variables. Furthermore, pCO_2_ and arterial glucose were not associated with any of the MD-variables in the univariate analyses. Arterial lactate and arterial glucose were strongly associated in the early phase (r = 0.38, *P*-value = .004) and the vasospasm phase (r = 0.39, *P*-value = .003).

In multiple linear regression analyses with MD-glucose as the dependent variable (Table 4), higher pO_2_ was independently associated with lower MD-glucose in the both the early phase and the vasospasm phase. Higher arterial lactate was independently associated with higher MD-glucose in the vasospasm phase.

### Arterial Blood gas Variables in Relation to Delayed Ischemic Neurological Deficits and Clinical Outcome

Patients who developed DIND during the first 10 days had similar ABG variables in the early phase and the vasospasm phase as those who did not develop DIND. Lower pCO_2_ in the early phase, higher arterial glucose in both phases, and higher arterial lactate in the vasospasm phase were associated with worse clinical outcome/lower GOS-E (Table 3).

## Discussion

In this multimodality study including ABG, ICP, and MD data from 60 aSAH patients, our findings indicate that the arterial content of variables that promote cerebral vasoconstriction (high pO_2_) were associated with better cerebral pressure reactivity, whereas those promoting vasodilation (high arterial lactate) had the opposite association. Nevertheless, higher pO_2_ correlated with less substrate supply, whereas higher arterial lactate had the opposite association. It is possible that aSAH patients with proximal large-vessel vasospasm exhibit a nearly exhausted distal vasodilatory reserve and by inducing cerebral vasoconstriction this reserve and consequently the cerebral pressure reactivity increase. However, cerebral vasoconstriction also reduces CBF and could negatively affect cerebral substrate supply. Larger experimental animal studies as well as clinical multimodal studies, including CBF imaging, are needed to validate this hypothesis and to determine how these ABG variables impact on CBF, cerebral oxidative energy metabolism, and clinical outcome.

### Arterial Blood gas Variables and Cerebral Pressure Reactivity, Blood Flow, and Energy Metabolism in Aneurysmal Subarachnoid Hemorrhage

In this study, we found that higher pO_2_ and lower arterial lactate were associated with better/lower PRx, but they also correlated with a more limited substrate supply. This contradictory finding may be explained by several mechanisms.

**Figure 2.**
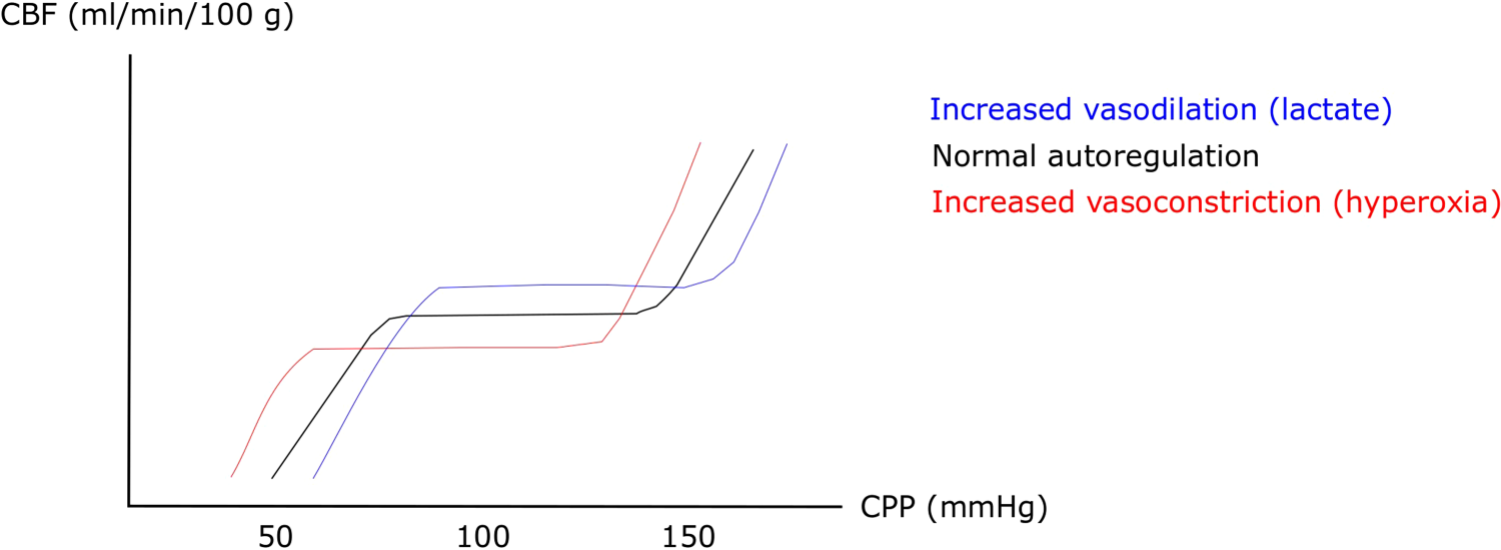
The lassen curve – effects of increased vasodilation and vasoconstriction. The figure demonstrates the Lassen curve of cerebral autoregulation. Agents that increase cerebral vasoconstriction (eg arterial hyperoxia) at a certain CPP, increases the vasodilatory reserve at that CPP level and leads to left-shift of the curve so that the CBF plateau is lower, but can be kept constant at a lower CPP than with lower arterial oxygen levels. An opposite effect could be expected with agents that predispose for cerebral vasodilation (eg hypercarbia and high arterial lactate). CBF = Cerebral blood flow. CPP = Cerebral perfusion pressure.

Patients with aSAH typically exhibit large-vessel vasospasm, which may be accompanied by a compensatory distal vasodilation to maintain a sufficient CBF. This gradually exhausts the vasodilatory reserve and leads to an impaired pressure reactivity and ischemic CBF (Figure 2). Variables that counteract distal cerebral vasodilation will increase the vasodilatory reserve and possibly improve pressure reactivity, but may also reduce CBF and the cerebral substrate supply. This modulatory effect of arterial variables on the vasodilatory reserve and pressure reactivity has been demonstrated in a previous study on pCO_2_, which showed that as hypercarbia reduces the cerebrovascular tone, the cerebral vasodilatory reserve becomes exhausted at a higher CPP/MAP and this shifts the Lassen curve and the lower limit of autoregulation to the right.^38^ Similarly, other arterial variables could also shift the Lassen curve by modulation of the cerebrovascular tone. For example, arterial hyperoxia decreases CBF at a certain CPP by cerebral vasoconstriction to keep brain tissue oxygenation constant,^11,12^ high arterial lactate increases CBF to meet metabolic demand,^27^ and hyperglycemia may disturb endothelial regulation of CBF by inducing vasodilation.^39^ Hence, our results, ie that higher pO_2_ and lower arterial lactate were associated with improved cerebral pressure reactivity, but lower substrate supply, are in line with that variables promoting cerebral vasoconstriction increases the vasodilatory reserve and pressure reactivity, but this could lead to decreased CBF and substrate supply. This is consistent with studies showing that arterial hyperoxia has been associated with increased risk of cerebral vasospasm,^14^ DIND,^15,16^ and unfavorable outcome^15,16^ in previous aSAH studies. However, the association with arterial lactate is much more complex to interpret, as it may both represent a certain underlying physiology and have an independent impact on cerebral physiology. Hence, previous studies on systemic lactate in aSAH have not been consistent, as some have found a positive^25^ and others no^40^ association between systemic lactate and DIND. Furthermore, we found no relation of the vasodilator high pCO_2_ and the vasomodulator glucose with cerebral pressure reactivity or the MD-variables. Possible explanations for the negative findings include a too small patient population and too tightly targeted ABG variables to detect an effect on the cerebral physiology.

Similar associations between ABG variables and cerebral pressure reactivity as in the current study have been found also in TBI. Variables promoting cerebral vasodilation (low pO_2_, high pCO_2_, high arterial lactate, and hyperglycemia) have been associated with worse pressure reactivity in TBI.^9,17,23,24,28^ The CBF pattern in TBI differs from aSAH, as cerebral hyperemia with concurrent intracranial hypertension is frequent in severe TBI.^41^ In these cases, systemic variables that increase the cerebrovascular tone could then potentially improve pressure reactivity and reduce hyperemia.

However, it is also possible that the ABG variables could influence the MD-variables by other mechanisms than via CBF. For example, arterial hyperoxia could increase energy expenditure and decrease the facilitated cerebral transport of glucose, resulting in a lower cerebral interstitial glucose.^42^ Arterial lactate may also increase MD-glucose by other mechanisms than CBF, as increased cerebral lactate delivery could be used as an alternative cerebral energy alternative and spare cerebral glucose.^26^ However, cerebral delivery of arterial lactate is relatively low at concentrations around 1–2 mM.^43^ There was no association between arterial and MD-glucose in the current study. This could be explained by that the MD-glucose level depends not only on arterial glucose levels, but also on CBF and the rate of glucose metabolism, and the relative contribution of the latter two could surpass the influence of arterial glucose levels. This is consistent with previous findings from our group, which showed that there is great variability interindividually and intraindividually over time in the association between arterial and cerebral glucose.^44^ It should also be mentioned that energy metabolic disturbances are not just a question of poor cerebral substrate supply. Changes in energy turnover and mitochondrial function are common and may cause cerebral energy failure.^45^ These pathomechanisms may also influence the association between arterial metabolic content and cerebral substrate supply.

### Arterial Blood gas Variables and Clinical Outcome

In this study, we found that higher arterial pCO_2_, lower arterial glucose, and lower arterial lactate were associated with better clinical outcome (higher GOS-E). Higher pCO_2_ is often more commonly employed in the absence of intracranial hypertension (although not correlated with ICP in this study) and may increase CBF and brain tissue oxygenation, which may explain the underlying mechanism for the association with better outcome in the current and in previous studies.^20,21^ Higher arterial glucose was associated with poor clinical outcome in the current study, similar to previous findings.^25^ Hyperglycemia could be a biomarker of pre-injury illnesses, post-ictal stress response, and reflect a more severe brain injury, but it is possible that hyperglycemia, per se, could induce secondary brain injury. Hyperglycemia has previously been associated with DIND,^25^ possibly by disturbing pressure reactivity^23,24^ and oxidative energy metabolism,^24^ although we found no support for these pathomechanisms in this study. High endogenous arterial lactate may reflect arterial hypotension, stress, and disturbances in renal/liver elimination, which may indicate a worse systemic state, worse brain injury, and more secondary insults from low CPP. In line with this reasoning, high arterial lactate was associated with poor outcome, similar to previous studies.^25^ However, it is possible that exogenous arterial lactate may increase CBF (possibly with worse pressure reactivity) and spare cerebral glucose. This needs to be determined in future studies. Arterial hyperoxia could increase the risk of cerebral vasospasm^14^ and DIND,^15,16^ and has been associated with unfavorable outcome in some,^15,16,46^ but not all studies.^21^ However, in this smaller study, we found no association of pO_2_ with DIND or clinical outcome.

### Limitations

First, this was a retrospective study with its inherent limitations of potential confounding variables. Second, we expected to detect an association between pCO_2_ and arterial glucose with PRx and the MD-variables. However, the patient cohort was small and the patients were treated in the NIC with vigilant monitoring and optimization of the ABG variables within targeted intervals. The net effect of these ABG variables on PRx and MD-variables might also likewise be small. This combination might have reduced the chances to detect an effect of the ABG variables on the cerebral physiology. Third, this was a single-center study of the subset of aSAH patients with the most severe acute brain injury who required multimodal monitoring. This limits the external validity of this study to the worst aSAH cases. Fourth, the MD measured cerebral energy metabolism in a small area of normal-appearing brain tissue in this study. Another MD location could have yielded a different result, considering that the energy metabolic status may differ between vascular territories and in penumbral areas close to an intracerebral hemorrhage. Fifth, the definition of DIND was based on clinical grounds as assessed during neurological wake-up tests. These tests are particularly sensitive to crude deterioration in wakefulness, language, and motor function, but to a lesser extent of alterations in eg cognitive functions. It is possible that secondary brain injury also occurred but were clinically silent in brain areas responsible for more complex functions including cognition.

## Conclusions

Higher arterial oxygen correlated with better cerebral pressure reactivity, but worse cerebral substrate supply, whereas arterial lactate had the opposite association in aSAH. This could indicate that agents that promote cerebral vasoconstriction improves an impaired vasodilatory reserve and thereby also pressure reactivity, but this could also lead to worse CBF as indicated by the impaired cerebral substrate supply. The net impact on oxidative energy metabolism was unchanged in this study and future, larger studies, including CBF imaging, are needed to better elucidate these associations. The study highlights the complex interplay between systemic and cerebral physiology in a severe brain disorder such as aSAH. Furthermore, it indicate that interventions that improves one variable could have unexpected effects on cerebral physiology, which should be considered in the neurointensive care of these patients. High-resolution multimodal monitoring could help in detecting and understanding these complex cascades. However, our results are hypothesis-generating and it is too early to implement these principles in clinical decision-making. Future experimental animal studies are planned by our group and a larger observation study is warranted to validate these findings.
